# Ethical reflections on end-of-life signs and symptoms in the intensive care setting: a place for neuromuscular blockers?

**DOI:** 10.1186/2110-5820-4-17

**Published:** 2014-07-08

**Authors:** Cédric Daubin, Lise Haddad, Dominique Folscheid, Alexandre Boyer, Ludivine Chalumeau-Lemoine, Olivier Guisset, Philippe Hubert, Jérôme Pillot, René Robert, Didier Dreyfuss

**Affiliations:** 1 SRLF Ethics Commission, la Maison de la Réanimation, Paris, F-75010 France; 2 Department of Medical Intensive Care, CHU Caen, service de Réanimation Médicale, avenue Cote de Nacre Caen, 14033 cedex, Caen, F-14000 France; 3 CHU Saint-Louis, Consultation Douleur, Paris, F-75010 France; 4 Institut Hannah Arendt, Université Paris-Est Marne-la-Vallée, Marne-la-Vallée, F-77454 France; 5 Department of Medical Intensive Care, CHU Pellegrin Tripode, Bordeaux, F-33076 France; 6 Department of Medical Intensive Care, Institut Gustave Roussy, Villejuif, F-94805 France; 7 Department of Medical Intensive Care, CHU Bordeaux Hôpital Saint-André, Bordeaux, F-33075 France; 8 Department of Pediatric Medical Intensive Care, CHU Necker Enfants Malades, Paris, F-75743 France; 9 Department of Medical Intensive Care, CH de la Côte Basque, Bayonne, F-64109 France; 10 Department of Medical Intensive Care, CHU Poitier, Poitier, F-86021 France; 11 Service de réanimation médicochirurgicale, CHU Louis Mourier, Université Paris Diderot, Sorbonne Paris Cité, UMRS 722, F-92701 Colombes cedex, F-75018 Paris, France

**Keywords:** Ethics, End of life, Critical care, Gasps

## Abstract

The death of a loved one is often an ordeal and a tragedy for those who witness it, as death is not merely the end of a life, but also the end of an existence, the loss of a unique individual who is special and irreplaceable. In some situations, end-of-life signs, such as agonal gasps, can be an almost unbearable “sight” because the physical manifestations are hard to watch and can lead to subjective interpretation and irrational fears. Ethical unease arises as the dying patient falls prey to death throes and to the manifestations of ebbing life and the physician can only stand by and watch. From this point on, medicine can put an end to suffering by the use of neuromuscular blockade, but in so doing life ceases at the same time. It is difficult, however, not to respond to the distress of loved ones and caregivers. The ethical problem then becomes the shift from the original ethical concern, i.e. the dying patient, to the patient’s loved ones. Is such a rupture due to a difference in nature or a difference in degree, given that the dying patient remains a person and not a thing as long as the body continues to lead its own life, expressed through movement and sound? Because there cannot be any simple and unequivocal answer to this question, the SRLF Ethics Commission is offering ethical reflections on end-of-life signs and symptoms in the intensive care setting, and on the use of neuromuscular blockade in this context, with presentations on the subject by two philosophers and members of the SRLF Ethics Commission, Ms Lise Haddad and Prof Dominique Folscheid. The SRLF Ethics Commission hopes to provide food for thought for everyone on this topic, which undoubtedly calls for further contributions, the aim being not to provide ready-made solutions or policy, but rather to allow everyone to ponder this question in all conscience.

## Introduction

It is the tension between the natural side of death and what death contributes to the meaning of life that is today at the heart of debates in France on the end of life. In some situations, end-of-life signs, such as agonal gasps, can be an almost unbearable “sight” because the physical manifestations are hard to watch and can lead to subjective interpretation and irrational fears. Ethical unease arises as the dying patient falls prey to death throes and to the manifestations of ebbing life and the physician can only stand by and watch. From this point on, medicine can put an end to suffering by the use of neuromuscular blockade, but in so doing life ceases at the same time. It is difficult, however, not to respond to the distress of loved ones and caregivers.

In this review the Ethics Commission of the Société de Réanimation de Langue Française (SRLF Ethics Commission) submit ethical reflections on end-of-life signs and symptoms in the intensive care setting, and on the use of neuromuscular blockade in this context, with presentations on the subject by two philosophers and members of the SRLF Ethics Commission, Ms Lise Haddad and Prof Dominique Folscheid.

## Review

### Part 1

#### The point of view of the SRLF ethics commission

##### End of life: a source of ethical tension

Progress in science and technology in recent decades, such as that made in medicine, has helped considerably to improve the living conditions and health of the populations of the industrialised countries, enabling people to reach a greater age and to live longer, quite often in full possession of their physical and cognitive faculties. Yet this progress has not intrinsically altered our perception of death, except to make it more unacceptable. Although death is seen as a natural phenomenon, part of the normal order of things, it is often an ordeal and a tragedy for those who witness it, as death is not merely the end of a life, but also the end of an existence, the loss of a unique individual who is special and irreplaceable. Death is thus experienced sometimes as an injustice, an outrage, whatever ideas, representations, and beliefs are held on what happens to the person after death, whether death is seen as annihilation or not, as an enigma, a mystery, a passage, a metamorphosis… For many people, whether or not death has meaning depends more on the sense, or lack of it, accorded to life, in accordance with their religious, spiritual or philosophical views. The perception of death differs according, for example, to whether life is held to have no meaning unless it is lived with full possession of all physical and mental faculties, or rather that it is part of a greater whole that includes, for example, an afterlife, which gives transcendent meaning to the hardships of the life that is coming to an end. It is the tension between the natural side of death and what death contributes to the meaning of life that is today at the heart of debates in France on the end of life. Medicine obviously is not immune to this tension. It responds to the tension through a form of prudential wisdom. Because medicine above all relates to human existences prey to diseases that may be seriously debilitating, life-threatening or life-ending. In the struggle for life, it is medicine’s task to preserve the conditions of a life sufficiently viable to give rise to a veritable existence, and not to seek to avoid death indifferent to the conditions of the existence that ensues. It was in this spirit that the SRLF made its recommendations of 2002 on the withholding and withdrawal of treatment in intensive care [1]. These recommendations were updated in 2009 [2] and were fundamental in the drawing up of Leonetti’s law on the rights of patients and the end of life (No. 2005–370 of 22 April 2005). In 2010 an end-of-life issues group was tasked, among other things, with reviewing the state of knowledge on end-of-life conditions and related healthcare practices and with bringing to the public debate objective and reliable data on the reality of end-of-life situations in France. Once again it was the same spirit that drove the recent public debate led by Professor Didier Sicard, Honorary President of the French National Ethics Committee, who was asked by the French President to review end-of-life issues, with a view to drawing up a draft law on the end of life and palliative care.

### Particular case of end-of-life signs and symptoms in the intensive care unit

The laws in place provide a framework sufficient to answer the vast majority of medical and ethical problems that intensivists are likely to encounter in end-of-life situations. It is important here to remember the recommendations of the SRLF [2], the Code of Medical Ethics (Article 38) and the Penal Code (Article 221–1), which stipulate that in end-of-life situations “the physician does not have the right to cause death deliberately”. Most of the time in intensive care, death occurs quite quickly once treatments are ineffective or are withdrawn because they are “futile”. In certain situations, withdrawal of treatment, particularly mechanical ventilation, can trigger gasping respiration, which can be a distressing sight, particularly as it may in some cases last for hours. End-of-life signs and symptoms pose a difficult problem, through how they are seen, interpreted and represented, notably in terms of possible suffering experienced by the patient. Faced with a patient prey to disturbing manifestations of a prolonged dying process, those present (caregivers and loved ones) are confronted by their own powerlessness and finiteness. And the attitude of the healthcare team is rarely unequivocal. So what is to be done? Shorten by any means, once all forms of sedation and analgesia have proven ineffective, the last convulsions, which are often seen as violent manifestations of a body locked in a final battle against certain and imminent death, when consciousness is doubtless no longer there? Or instead respect this ultimate moment of life, this unique and singular time that everyone should be able to live through, without having their last moments of life purloined, whatever the cost to loved ones and caregivers?

In this context, Perkins and Resnick [3], in an article published eleven years ago, clearly posed the question of the use of neuromuscular blockade to end the most extreme end-of-life signs, the gasps of agonal respiration, which are a source of intolerable distress for loved ones. Perkins and Resnick’s article followed on from previous publications which questioned the use of muscle relaxants in the practice of withdrawing ventilation from mechanical ventilator–dependent patients for whom the decision has been taken to stop intensive care [4–6]. In these earlier publications, the authors firmly condemned the use of muscle relaxants (devoid of sedative or analgesic effect) initiated just before withdrawal of mechanical ventilation with the sole aim of suppressing impending terminal signs, so as to give the appearance of a calm and serene patient. They deemed this practice ethically indefensible and likened it to a form of euthanasia. The authors reaffirmed their opposition to intention to cause death. They even proposed that, for patients already under neuromuscular blockade for therapeutic reasons when the decision is taken to withdraw care, the administration of muscle relaxants should be stopped and their effect allowed to wear off completely before withdrawing ventilatory support, so as not to jeopardise any chance of survival the patient may have. On the other hand, the same authors did not exclude the possibility of withdrawing mechanical ventilation if paralysis persists after discontinuation of neuromuscular blockers, in the single case where the probability of the patient surviving withdrawal of ventilation, in the absence of neuromuscular paralysis, is reckoned to be nil. Whatever the context, the authors stressed the importance of suitable sedation-pain relief. However, the problem posed by prolonged gasping after withdrawal of ventilatory support was not addressed.

Perkins and Resnick [3] report the cases of two young patients, notably that of a 14-year-old girl with a neuromuscular disease who was admitted to intensive care because of respiratory distress following pneumonia and was mechanically ventilated at the express request of her parents. Three weeks later, faced with the patient’s complete dependence on mechanical ventilation and her clearly and repeatedly expressed refusal of ventilatory support, and after numerous discussions with those concerned, the medical team decided to remove ventilatory support and the patient was extubated. She died the same day surrounded by her loved ones, but before dying she experienced gasping respiration for 13 minutes despite increasing doses of morphine. At a meeting several months later, the mother said that she wished she had not seen her daughter gasping, that she was convinced that her daughter had suffered and that in her dreams she frequently relived her daughter’s last unbearable moments.

In the other case a man of 18 had an untreatable neurological disease manifesting as severe dystonia and spasms so severe that endotracheal intubation was performed to palliate respiratory distress. Treatments (baclofen, benzodiazepines, narcotics) failed to control the spasms and prevented ventilator weaning. The parents subsequently asked for mechanical ventilation to be stopped. After extubation, aggressive use of baclofen, benzodiazepines, narcotics, and barbiturates controlled the muscle spasms and respiratory obstruction. But these treatments proved ineffective once gasps started. The patient was in a coma and had apnoea for 30 to 60 seconds, interrupted by agonal breaths, for 40 minutes. The father repeatedly asked: “Isn’t there anything else you can give him? He is suffering”.

The authors therefore re-examine an essential ethical principle of medicine enunciated by Hippocrates: “the physician does not have the right to induce death deliberately” (Article 38 of the Code of Medical Ethics and Article 221–1 of the Penal Code) and propose that, in certain circumstances, in well-sedated patients who have received well-managed palliative care but in whom agonal gasps are intractable, that the administration of muscle relaxants can be envisaged to suppress the gasping response if it is poorly tolerated by loved ones, so as to ensure a “serene” death. This position elicited numerous reactions against the use of muscle relaxants to suppress gasping [7, 8]. However, among the many arguments advanced by Perkins and Resnick, two are particularly worthy of our attention since they lie at the heart of the dilemma facing caregivers. The first relates to what a patient may or may not feel in the gasping phase of the dying process, while the second concerns the obligation felt by caregivers to alleviate the distress experienced by the patient’s loved ones for whom the suffering perceived in the gasping respiration becomes intolerable.

### Is the patient suffering during agonal respiration?

Gasping respiration is the last phase in the dying process. It is irreversible and always followed by death. Before this, consciousness may fluctuate and the patient may or may not alternate between phases of coma, confusion, or agitation. Sensory perception is usually preserved and, if he can still communicate, the patient may suffer from visual or auditory hallucinations. There may be laryngeal stridor, bronchial rales, or excessive respiratory secretion. Respiration is more or less effective, blood pressure varies, and severe diarrhoea may occur, indicating ischaemia of the digestive tract. All of these signs should be treated appropriately, or better still anticipated and prevented by specific care [9]. Preterminal gasping signifies that death is imminent (hours or minutes). This singular moment is defined by a deep coma, accompanied by signs of decerebration, decortication, and loss of perception. Consciousness is lost and there are no longer signs of emotion [10, 11]. The observed clinical signs, such as myoclonic jerks and gasps, which are also described as terminal apnoea, are linked to cerebral anoxia. Gasps are therefore a physiological response to hypoxaemia. They are reflex movements common to all mammals that originate in the medulla of the central nervous system. Gasping is an auto-resuscitative mechanism inasmuch as it increases venous return and cardiac output, and raises aortic pressure as well as coronary perfusion pressure [12]. In various animal species, gasps only occur with deep hypoxaemia when the partial pressure of oxygen drops to below 5 to 15 mmHg. Gasping indicates approaching death. Scientifically, everything suggests that gasping patients do not feel pain or respiratory discomfort since clinically there is no objective evidence of residual consciousness. Some authors consider that all medications, including sedatives and analgesics, are useless and disproportionate at this stage of the dying process [10]. It is also admitted that increase of the analgesics and sedatives usually administered to patients in the terminal phase in intensive care to a dose sufficient to abolish all pain, and perhaps even consciousness, would not suppress the more dramatic manifestations of gasping. Only muscle relaxants can suppress gasping respirations [3, 8]. It is clear that at this stage of the dying process, medication offers nothing and should give way to support not only of the dying patient, but also of the patient’s loved ones.

### Gasps in the terminal phase as witnessed by loved ones and caregivers

This moment of the death process is always hard for loved ones, but also for caregivers. It starkly confronts everyone with the realities of finiteness and death. It is a source of tension and anxiety exacerbated by interpretation of agonal respiration, by projections, and by the meaning that the onlookers give to the death scene played out before their eyes. Even more complex and troubling is that end-of-life signs (such as tears), which are only bodily reflexes, were a few instants before an expression of pain, of discomfort, of sadness or suffering that justified the therapeutic act. In the continuum of the dying process and the care that accompanies it, how can we be certain that the observed loss of consciousness and perception is a sign of entry into the terminal phase or of the effectiveness (excessive even) of sedation-analgesia? The occurrence of gasping, however, should not raise doubts in the clinician’s mind. An imperative for all caregivers seeking to make the unbearable more tolerable is to explain, describe, and inform so as to shed light on this demise and to inscribe it in the natural process of life. In this context, the perception by loved ones of the feelings of caregivers regarding gasps, of a perceptible awkwardness of personnel ill at ease or on the contrary more “supportive” and able to speak of what is happening as a troubling but natural phenomenon, would also contribute to a greater acceptance (or not) of these end-of-life signs [13]. Likewise, the words used to describe gasps—like the “agony of agonal respiration”—should be chosen carefully as they have highly negative connotations and are associated in our minds with something greatly painful suggestive of suffering. Whatever means the health care team employs to render the intolerable more tolerable, there remain situations that are harder than others, and families and caregivers who cannot be comforted. For the time of dying is not experienced in the same way by loved ones and health care teams, particularly if it is prolonged. Whereas most caregivers see dying as natural, conscious that it proceeds through different phases and lasts as long as it must, for loved ones dying is felt to be interminable, an eternity all the more hard to endure as for them it makes no sense. How can caregivers not respond to the distress of families for whom the suffering experienced on witnessing prolonged gasping is added to the suffering associated with the loss of a loved one? Do they not have, moreover, an ethical duty, stated by Perkin and Resnik [2] using the American Institute of Medicine's definition: “A decent or good death is one that is: free from avoidable distress and suffering for patients, families, and caregivers; in general accord with clinical, cultural, and ethical standards?” [14].

### Ethical questioning

Ethical tensions arise from the fact that the physician cannot alleviate the dying patient’s gasping respiration, except by relieving it and at the same time curtailing the life he is witnessing. Medicine is therefore stumped, inoperative, and yet finds it hard not to respond to what has become an unbearable suffering of loved ones, and even of caregivers, who witness the last convulsions of one of their own in whom they have invested much. In other words, the ethical problem raised is that of a shift in ethical aim, which initially targeted the patient and now focuses on loved ones. Does such a rupture relate to a difference in kind or a difference in degree, given that the dying patient remains a person who cannot be reduced to a thing, despite appearances, a body which continues to lead its own existence, to manifest life by movement and sound? How then to respond when loved ones beg for something to be done to end the signs that they find unbearable and which they often think must be intolerable for the person from whom they emanate?

At the stage of persistent gasping, only a muscle relaxant can act effectively on rales, hiccups and convulsions and bring to an end the sounds and contortions of a body struggling against inevitable death. Envisaging the possibility of neuromuscular blockade invites us to question our intentions. Do we wish to control the uncontrollable, to preserve mastery over a natural process, over an eminently human moment that goes beyond the medical and which escapes our grasp? Even if it means denaturing it, on the pretext of wanting to bring relief to loved ones, to medical staff, or to oneself? Is it a symbolic wish to sacrifice the dying person who is already no longer seen as who he was, but instead as neither really alive nor dead, neither body nor corpse, in a somewhat utilitarian attempt to procure less disquiet for the greater number? Laudable though it may be to seek to comfort those who live through the tragic experience of the death of a loved one, is not the use of muscle relaxants in the terminal phase also a way to spare us from thinking about dying and especially its clinical implications? Neuromuscular block used to avoid the confrontation with death, as if to escape death, in a way to die without dying, that is to die as we fall asleep, soundlessly, without signs, without excess, discreetly. Is it not on the contrary the last duty of the physician to preserve what the dying man would consider his dignity, the dignity of not being seen in this frightful state, and also to honour the commitment he has made to dying man’s loved ones to ensure a peaceful death without suffering?

As there are no simple and unequivocal answers to these questions, the SRLF Ethics Commission thought it would be interesting to ask two thinkers who are members of the SRLF Ethics Commission, Ms Lise Haddad and Professor Dominique Folscheid, to share their thoughts on terminal signs and symptoms, and in particular the use of muscle relaxants.

### Part 2

#### The point of view of Ms Lise Haddad, philosopher and member of the SRLF ethics commission

In intensive care, in hospital, where death most often occurs, how can we provide both medical and humane support for the dying?

The end of life, a periphrasis used to suggest expressions with greater connotations, such as demise or death struggle, should pass like the rest of life, gently, peacefully, comfortably and with dignity, as far as is possible. The difficulty resides in the status of the dying or terminally ill patient: hovering at the fuzzy border between life and death, we no longer know very well with whom we are dealing—a patient, someone moving towards death? How can we qualify the person’s presence, which is both verifiable and precarious, fleeting, evanescent? How can we establish a relationship with someone who no longer seems conscious or is plunged into a state of altered consciousness, who no longer speaks, who no longer exchanges looks or gestures?

With the competitive resonances of the etymology of the word agony (Greek *agōniā*, contest), the death struggle, the dying person seems to be engaged in a battle against an invisible enemy, oppressive and torturing: respiration becomes increasingly hoarse, irregular and difficult, the body seems to have lost all autonomy and is shaken by uncontrolled jerking and twisting. No longer accessible for an exchange, the dying person offers those who loved him, the medical team who cared for him, the physicians who tried to save his life, a terrifying spectacle which confronts everyone with their doleful powerlessness.

Between life and death, the dying person seems doomed to complete solitude, and at the same time experiences his death in an absolutely singular way. In him is embodied the unique figure of death, his death. He denies the abstract and hence reassuring character of human finiteness. The circumstances of death form the last manifestation of individuality. The end, in a precise context, at a certain time, with its rales, its hiccups, its shudders, happens all the same in a specific way. The determined form of a person is dimmed and undone, pointing to imminent oblivion. “The truth is not death […] only its foreshadowing” explains Bataille. And Didi-Huberman [15], a contemporary French philosopher and specialist in aesthetics, who quotes Bataille, explains that the violence is not in death, “which would lead to the annihilation of violence itself”. It is not death that is the most dreadful, but “only its symptom” […]. “A *something*, an *anything*, notably visual, to which is briefly attached the thing lost, the thing to be lost”. In this same work, Didi-Huberman, speaks of being engaged in “a *work of forms* equivalent to what would be a *work* of childbirth or of death”. This reflection on a new way to address the work of forms that Bataille presents in his journal *Documents,* according to Didi-Huberman, amounts “to implementing the alteration and declassification of the *aesthetic* (which is called taste) into the *aesthesic* (which is called desire, pain, disgust), and of the *symbol* (shareable) into *symptom* (intractable)”. Here reappears the very source of the aesthetic in its etymology signifying feeling, perceiving. The violence visited upon forms, the disfiguring, again reveals the origin of the aesthetic feeling in pleasure or sorrow, whence the malaise experienced by the onlooker.

If it is possible to draw a parallel between the aesthetic, that is the field of reflection on art, and the potential treatment of the death struggle, it is because the person we seek to treat with muscle relaxants is no longer the patient assumed to lack all feeling, or at any rate to be incapable of showing it, but the witness, in other words the spectator.

This transition of the patient, who is entering death, raises questions in the minds of those who witness this demise. What are we treating? Do we imagine we are answering the patient’s wishes by acting on the form of his body at this ultimate moment? Are we replacing him by his loved ones who will preserve and care for his memory since they now become the sole interlocutors? Are we also responding to the frustration of the caregivers in the face of the failure of resuscitation, of “reanimation”, while avoiding a bed occupancy that is as pointless and costly as it is distressing? In any case, neuromuscular block precipitates this difficult and elusive transitional moment and fixes it in a stiffness that is soothing for others. This question is perhaps also to be found at the heart of debates on euthanasia: in the search for a dignified death is outlined the dream of death mastered, formal, retaining a social, a shareable dimension.

The unbearable resides in what is seen: the loss of form in convulsions, the transformation of articulate and meaningful words into rales and other organic noises, the end of the dying person’s control over his body. All death signifies the end of an irreplaceable individual, the collapse of all promise that he represented, the infinitely painful separation from loved ones. It is not a question of contesting the heart-rending nature of this moment. Death cannot be avoided: sooner or later, here or elsewhere, it will happen. What then are we seeking to mask with neuromuscular block, that is by inducing mortal paralysis, since we suppose that the patient is beyond all suffering and anguish, which is exactly what is enabled by neuromuscular block, while it would be very hard to imagine the same for a conscious person?

From the moment when the patient is no longer thought of for himself, but rather as an object of representation, the landmarks of aesthetic theory are of help in our attempt to interpret this intolerance of the sight of the death struggle. More than the patient’s relation to his own death is now the meaning that this demise assumes for those who become its witnesses. Walter Benjamin, the German 20th century philosopher, in his book *The Origin of German Tragic Drama*[16], explains how baroque drama stages the sorrowful as opposed to the tragic. The tragic shows the suffering of the hero. The silence of his death reflects his solitude, and his sacrifice, “which establishes a new law while respecting the old”, elicits the respect and recognition of theatre-goers, who represent the community and are invited to take part in this confrontation of value systems. The German baroque stage-form called *Trauerspiel* suggests through its root *Trauer*, mourning, a whole funereal, ostentatious universe. The spectators no longer are the judges of a situation embodied by the hero, rather they become the point of departure of the drama: “*Trauerspiel* must be understood from the viewpoint of the onlooker. He sees onstage situations imposed upon him, an interior place of feeling. Instead of meaning, he is plunged into the world of feeling by the staging of the funereal, the insistence on sensitivity, like the inarticulate accents of a wail. The function of baroque writing full of imagery is less the drawing back of the veil than almost the stripping bare of sensitive objects”. The death of the hero in tragedy underscores the exaltation of the individuality of the hero who, through his acceptance of death, calls into question the system of values that crushed him. His death then takes on an abstract meaning, whereas the insistence, in the baroque, on sensitive manifestations has the purpose of reproducing the universe of feelings, which is neither purely theoretical nor wholly irrational. The body becomes the stage on which is acted out this conflict between reason and the irrational. Benjamin refers moreover to Descartes’ theory of the passions according to which the mind, pure reason, is subject to the violence of the affects through the intermediary of the body. Thus, baroque drama, in presenting onstage the corpses of the characters, does not allude to immortality, but on the contrary to their “future as corpses”.

The emphasis of the stigmata of dying in baroque art, the insistence on the funereal, the recurrence of appalled eyes in pictorial representation, tend to produce an effect on viewers and to illustrate a theory of passions, by stressing not the sense of a death assumed, but dispossession of self by the body.

This parallel drawn between a medical reflection and the theory of the art shows, in this hesitation about whether to allow or to mask the signs of impending death, a sort of shift from the symbolic to the real. The sight of the dying process and of death is not used in a representation but rather is staged directly on the patient. Everyone can choose a symbolic reading of the event when confronted by a painful situation, but here the work is not done on the symbolic, but operates a kind of short-circuit. The body itself projects an image of serenity through an outside and artificial intervention with no soothing effect on the patient’s feelings. This spiriting away of death has without doubt the objective of attenuating the suffering of the patient’s medical team and family and friends (this conflation creates the rest of the problem). But how far can we protect others from the terrible impact of the death of a loved one?

What authorises an act that no longer has any therapeutic purpose, nor even is intended to improve the patient’s comfort? The risk lies in possible subreption by physicians. Indeed, acting on physiological signs presupposes scientific knowledge, or as Alfred Baeumler [17] explains in *The Problem of Irrationality in the Aesthetics and Logic of the XVIIIth century*, “As a subject of science, he [man] is no longer the living and real being, but only a theoretical being. […] subject to the general laws of the material world”. According to Baeumler, the only domain where man is recognised as himself, as a “living individuality”, is aesthetics, because he is only conscious of his uniqueness in the world of feelings. As it is effected by physicians at a moment they deem opportune, medical action intended solely to give form to a body in death is an aesthetic preoccupation with scientific methods in a person who is still living when the muscle relaxants are administered. In a sense, by way of concern for tactfulness, the purpose is to mask the truth of what is happening: the end of life of an individual, with no benefit for the dying person.

Neuromuscular block at the end of life seems then to raise several questions: that of the switch from concern for the patient to concern for his family and friends, and in this shift that of the instrumentalisation of the patient as an object gazed upon; that of the legitimacy of physicians acting upon the patient solely to soothe the feelings of the patient’s family and friends. The physicians’ interest is then concentrated on what is seen of the patient’s body, by a shift from the symbolic to the real: they attempt to anticipate and soothe the pain of grief by masking the beginnings of the loved one’s demise.

### Part 3

#### The point of view of professor Dominique Folscheid, philosopher and member of the SRLF ethics commission

##### Dying and gasping. For third party ethics?

What is a physician ethically permitted to do when a dying patient experiences gasping respiration? In describing the situation, there can only be negatives. No longer is it a question of curing, of limiting or stopping treatments, of providing palliative care or support. And available forms of sedation are unable to curb the violence of the reactions that occur. From a medical point of view, we are in the midst of certainty and uncertainty: certainty that the end is near, that the patient is unconscious, that his condition will not progress to a lasting coma, virtual certainty that he is not suffering; yet also uncertainty about the precise moment of death, if it exists, and uncertainty about the status of the patient who is gasping.

### What status?

The words are already suggestive. The patient is no longer dying but is engaged in the death struggle, the agony. Agony means a violent struggle or combat, the struggle that precedes death, from the Greek *agōniā*, derived from the verb *agein* (in Latin *agere*), which means to lead, to drive before. The same Indo-European radical *ag* is found in to agonise, but also in agenda and agitation.

The dying person then is a fighter. We say of someone whose life is virtually over that he is struggling with death. Also that a battle ends because there is no one left to carry on the fight. But when the battle does not end, who then is the combatant? In short, in the case of gasping, is there still someone, a person, who is struggling?

How then can we repeat, with Epicurus, that: Death is nothing to me, since while I exist, my death is not, and when my death occurs, I do not exist? A formula that is flawed in three ways in the case to hand. First, because the formula’s function, which is to free us of the anxiety of death, is foiled by the gasping. Second, because the “I” that is pivotal in the formula seems to have disappeared. Third, because the either/or formulation, which makes the line between life and death razor-sharp, a tipping point outside time, devoid of temporality, seems to give way to a process that can be spread over time, creating a grey area. An area that will for ever remain a mystery (rite known only to the initiated), because there is no initiate (Greek *mystēs*), no conscious subject capable of surviving such an experience and describing it to us afterwards.

Reconsidering the formulation in terms of Jankélévitch’s “between two”, we could say that we are between an “already no longer” (death has done its work) and a “not yet” (death has not reached completion). Something between post-death and “cadaverisation”. Recall what Aristotle said of the cadaver: it is no longer really a body, except in a manner of speaking, because the soul has flown, the soul being the form of the body, and so the matter that comprises the body is left to itself, leading to the disintegration and decomposition of its elements.

Here insight is to be found in Leibniz’s belief that a simple substance can only vanish through annihilation, a composed substance only by decomposition. This offers an intellectual framework for modern scientific descriptions according to which we die, so to speak, in parts, organ by organ, the stem cells surviving well after death. Respiratory gasps can then be considered as forms of powerless revolt, the desperate struggle of certain parts of the body at a subordinate level—archaic reflexes.

Consider now what is the death of a human being. Even if called “natural”—which it is, in the end, in all cases, whatever its external and occasional causes—it cannot be reduced to what happens in all other living beings strictly subject to the cycle of nature, which concatenates life and death within a species. As Heidegger said, strictly speaking only man dies, since the animal perishes. In fact, the death of a human being is twofold. On the one hand, the most important in my eyes, it is a historical event: the demise of a singular being, unique and irreplaceable, hence a tragedy. On the other hand, death is the normal outcome of the life of a biological individual, mortality being an integral part of its natural quality or character. Which gives us a duality without any dualism, because while alive a human is one with his body. Which does not prevent us, quite the contrary in fact, from considering our body in two distinct ways: the organic body (German *Körper*), and the lived body, as experienced by the person (*Leib*).

### The ethical relation: from two to three

In our uncertainty over the status of the gasping patient, we can begin by taking two reference points, fixed and certain. First: however long a terminally ill person is dying, ie, is still alive, conscious or not, the relations others have with him are interpersonal. In the words of Buber, the relations concern the “I and Thou” in the case of the patient and his loved ones, the physician and the caregivers, plus the relation with the “It” (of “I and It”) of the patient which is the subject of health care, and so the organic body. But for everyone, the person is indeed there, indissociable from his body, whatever its condition, and ethics must govern the relations that we maintain with the person. Second: once death has done its work, we slip into a quite distinct issue. The person has been erased, leaving just a corpse which is no longer really a body, but a pure “It”, reduced to a thing among the things of the world. Just good for anatomic and pathologic study, for sampling, for whatever else. Logically, ethics becomes mute. And yet we find shocking and inadequate the response of Heraclitus, who advised the Ephesians to throw corpses out with the rubbish. For while a corpse is no longer a body as such, since it has been deserted by its form, it remains one for others.

A distinction has to be drawn between two sorts of forms: the animating principle (Greek *morphē*), which has disappeared, and the external configuration (in Greek *schēma,* in German *Gestalt*), which is maintained. The corpse is not then someone’s waste, but his mortal remains, that is his external configuration, residual but devoid of its animating principle, hence of life. The term “remains” is suggestive of the skin shed by an animal that moults. It is, of course, no longer the body itself or the flesh, but it still bears the traces of flesh and is seen as such by others. For what makes a person’s humanity is in very large part the relations he had with other people, elements intertwined inseparably with the physical body, while the person was living. What subsists of the body which is no longer a body is therefore paradoxically the flesh, for the investment that constitutes it is also ours. The family, loved ones, friends, then in a way become repositories and bearers of a part of the flesh of the deceased, in his stead. The deceased moreover keeps his name, his family ties, and still permits the forging of a memory of a singular history. This is why we honour a dead person, render homage to his virtues and actions, take care of his mortal remains, restore them after medical sampling and autopsy.

What can we take from these considerations of a dying person who is gasping? Once the normal means of calming the violence have been exhausted, one can obviously let things run their course and wait. But the situation can be intolerable for loved ones, caregivers, the physician himself. Unbearable in human terms, by mimetic projection onto the patient. Morally unbearable to see a human being reduced to a subjectless reflex system, to witness the vain revolt of organs against the body, nature rising against herself without for all that inventing a way, albeit diminished, to continue to live. In this situation, loved ones, caregivers, physician become “protagonists” in the strict sense, thrust forward and called upon to provide an answer, if possible.

Is it then ethical to try to humanise this death? We are not speaking of euthanasia, which consists in hastening the death of a living person. Nor are we speaking of rendering life artificial in order to delay an inevitable death. We are already in the orbit of death, and what we can still do there can be understood as a means of combatting death’s own waywardness and restoring its naturalness, which is the very definition of medical action. Except that here we do the reverse of what we would do ordinarily, which is to preserve life. By acting on an “It” which has become wayward we compensate for the incapacity of the dying person. A third party, the physician, then takes the place of the “I” who is absent but still treated as “Thou”. We are, it is true, only speaking about signs, but medicine does not give up treating signs, and uses aspirin, antidepressants, without treating the root of the problem. By using muscle relaxants we are clearly not in the doctrine of double effect, but rather in the single effect, since the harmful effect (death) is already at work. This would be the reverse double effect: the worst having happened, it is made less bad.

But there is more, if we take into account others, those who support the gasping person: loved ones, caregivers and even the physician, all overwhelmed by the unmanageable violence of these terminal signs. One may well wonder what medicine can do here, for none of these other people is ill in the sense of disease. They are though “suffering”, a term that Viktor von Weizsäcker preferred to “illness”. Indeed they suffer in two ways—in the form of illness, the subjective suffering, and sickness, the social suffering. As henceforth the sole bearers of the deceased’s flesh, in a way they become its substitute. Different from the deceased, yet the same.

In drawing here on Levinas, we are concerned with third party ethics, for if we consider just the “I-Thou” relation, that of the physician with the patient, we risk forgetting our obligations to others. This is a primary form of justice which is an integral part of the ethical relation. This is indeed an ethical issue, and not one involving free and equal subjects entering into a contract, since the persons confronted by the gasping patient are not only vulnerable, but also “vulnerated”, wounded. The physician is therefore ethically obliged. As Levinas said, “the other’s need, and not his freedom, obligates me in my freedom”.

Perhaps one day it will be proved that a patient in this state is already dead. Since the invention of brain death, everything seems possible. If this were to happen, muscle relaxants would pose no problem. Medicine would be nothing more than embalming the patient, but would nonetheless fulfil its role towards others.

In the uncertainty that remains, we find ourselves in a situation very close to that which prevails in terminal sedation. The link between the two would then be sleep, the person’s last sleep. From an ethical standpoint, there is nothing shocking about this since it would readmit an exceptional situation into the human community. All the deceased, whatever the manner of their death, find peace in this type of sleep. Let us not forget that the word cemetery comes from the Greek *koimētērion*, meaning “sleeping chamber”.

## Conclusions

The SRLF Ethics Commission hopes to enrich the thoughts of everyone on a subject that without any doubt calls for other contributions, the aim here not being to provide ready-made solutions, or even orders, but to empower everyone to think in all conscience.

## Author’s contribution

CD, LH and DF drafted the manuscript. AB, LCL, OG, PH, JP, RR, DD revised the manuscript for important intellectual content. All authors read and approved the final manuscript.

## Author’s information

All authors are member of the SRLF Ethics Commission, La Maison de la reanimation, avenue Claude Vellefaux, 75010 PARIS.

## Abbreviation

SRLF: Société de Réanimation de Langue Française.
